# Identification and validation of four photodynamic therapy related genes inhibiting MAPK and inducing cell cycle alteration in squamous cell carcinoma

**DOI:** 10.3389/fonc.2022.946493

**Published:** 2022-08-04

**Authors:** Yingchao Zhao, Jianxiang Dong, Yuxuan Liao, Hongyi Wang, Dawei Zhou, Jian Kang, Xiang Chen

**Affiliations:** ^1^ Department of Dermatology, Third Xiangya Hospital, Central South University, Changsha, China; ^2^ Xiangya School of Medicine, Central South University, Changsha, China; ^3^ Graduate School of Peking Union Medical College, Chinese Academy of Medical Sciences and Peking Union Medical College, Beijing, China; ^4^ Department of Dermatology, Xiangya Hospital, Central South University, Changsha, China

**Keywords:** cell cycle, MAPK, photodynamic therapy, cutaneous squamous cell carcinoma, A431 cell

## Abstract

**Introduction:**

Cutaneous squamous cell carcinoma (cSCC) is the second most common skin cancer, and photodynamic therapy (PDT) is a promising modality against cSCC. This study investigated the impact of PDT on the MAPK pathway and cell cycle alternation of cSCC as well as the related molecular mechanisms.

**Method:**

Expressing mRNA profile data sets GSE98767, GSE45216, and GSE84758 were acquired from the GEO database. The functions of differently expressed genes (DEGs) were enriched by Gene Ontology (GO) and Kyoto Encyclopedia of Genes and Genomes (KEGG) analysis. Least absolute shrinkage and selection operator (Lasso) analysis were used to establish a diagnosis model based on GSE98767. A correlation analysis and a protein–protein interaction (PPI) network were used to evaluate the relationship between cSCC-PDT-related genes and the MAPK pathway. Single-sample gene set enrichment analysis (ssGSEA) was performed on GSE98767 to estimate MAPK activation and cell cycle activity. Finally, the effect of MAPK activation on the cell cycle was explored *in vitro*.

**Result:**

Four cSCC-PDT-related genes, DUSP6, EFNB2, DNAJB1, and CCNL1, were identified as diagnostic markers of cSCC, which were upregulated in cSCC or LC50 PDT-protocol treatment and negatively correlated with the MAPK promoter. Despite having a smaller MAPK activation score, cSCC showed higher cell cycle activity. The PDT treatment suppressed the G1 to G2/M phase in JNK overexpressed A431 cells.

**Conclusion:**

CCNL1, DNAJB1, DUSP6, and EFNB2 were identified as potential PDT target genes in cSCC treatment, whose potential therapeutic mechanism was inhibiting the MAPK pathway and inducing cell cycle alternation.

## Introduction

Cutaneous squamous cell carcinoma (cSCC), a malignant tumor originating in epithelial cells, is the second most common skin cancer, with an increasing incidence in elderly individuals (1). Surgical excision is the first-line treatment for common primary cSCC (2), but the obvious scars resulting from extended resection, especially those on eyelids, ear lesions, or lips, lead to poor cosmetic outcomes. Moreover, patients on immune suppressants or anticoagulants and elderly individuals with comorbid conditions are ineligible for surgery. Nonsurgical approaches such as radiotherapy, chemotherapy, and cryotherapy are applied to the aforesaid patients. Unfortunately, these treatments are associated with severe side effects, chemoresistance, or a high recurrence rate (3). Thus, there is an urgent need to develop a novel therapy with a minimally invasive nature, good anti-tumor effect, and limited side effects.

Photodynamic therapy (PDT) is a burgeoning, safe, effective, and affordable remedy that provides a great cosmetic outcome for cSCC. PDT requires three components: a photosensitizer, light, and molecular oxygen. In the treatment process, the photosensitizer is first coated or injected into the tumor tissue, and then the nidus is irradiated by the light at the appropriate wavelength and energy. The photosensitizer in tumor tissues can produce a photochemical reaction to generate reactive oxygen species (ROS), which will react with bioactive components in tumor cells to kill them (4). Additionally, ROS can be rapidly metabolized in normal tissues around tumors, which signifies that PDT is highly selective for treating cSCC.

Previous mechanism-based studies have revealed that PDT induces apoptosis or necrosis of cSCC cells primarily through the direct cytotoxicity of ROS, complete occlusion of microvasculature that nourishes cancer cells, and stimulation of anti-tumor immunity (5). Furthermore, increasing evidence has shown that the occurrence and progression of cSCC may be related to abnormal regulation of mitogen-activated protein kinase (MAPK) signaling. The mutation of the tumor suppressor genes TP53, CDKN2A, NOTCH, and oncogene Ras is common in cSCC (6–8). The mutation of Ras leads to the declining activity of GTPase-activating proteins (GAPs), suppressing the transformation from active guanosine triphosphate (GTP)-bound states to inactive guanosine diphosphate (GDP)-bound states (9). Redundant GTP causes Ras activation and upregulation of downstream PI3K/AKT/mTOR and MAPK intracellular signaling (10) and eventually leads to excessive proliferation of cells and tumorigenesis. The best-known MAPKs include c-Jun amino-terminal kinases 1 to 3 (JNK1 to 3), extracellular signal-regulated kinases 1 and 2 (ERK1/2), ERK5 and p38 (α, β, γ, and δ) families (11). ERK is related to cell growth and differentiation, while JNK is related to inflammation and apoptosis. Previous studies (12) have proved that the suppression of JNK-dependent apoptosis, cooperating with paradoxical ERK activation, induces cSCC. To sum up, the progression of cSCC is closely associated with MAPK signaling (13). Moreover, some studies (14, 15) indicate that MAPK activation stimulates cell cycle transitions, and numerous therapies have exerted their anti-tumor effects by blocking the MAPK signaling pathway and thus arresting the cell cycle transformation. A recent study (16) reported that modified 5-aminolevulinic acid photodynamic therapy could suppress the ERK pathway, activate the JNK pathway, and inhibit cell proliferation and Cyclin-D1, which performed a regulatory function during the transformation from G1 to G2/M phase. Accordingly, we can infer that PDT probably affects cell cycle alterations in cSCC through the MAPK pathway.

Preceding bioinformatics analysis suggested that the combination of PDT and chemotherapy could upregulate ROS generation, blocking the cell cycle and proliferation (17). Besides, it seems that PDT downregulates the pathways related to tumor progression, metastasis, and invasion. Up to now, there are still rare reports about the comprehensive mechanism of PDT for cSCC treatment. We were interested in exploring whether inhibition of cSCC proliferation by PDT was related to MAPK signaling and its regulation of the cell cycle. In this study, we found a potential molecular mechanism of PDT for treating cSCC was that PDT inhibited MAPK activation, which was probably because the downregulation of JNK was greater than the upregulation of ERK. We preliminarily confirmed that the inhibition of MAPK suppressed the transformation from G1 to G2/M phase in cSCC using bioinformatics analysis and flow cytometry. Additionally, CCNL1, DNAJB1, DUSP6, and EFNB2 were upregulated in cSCC or LC50 PDT-protocol treatment based on our results. Thus, we speculated that increased CCNL1, DNAJB1, DUSP6, and EFNB2 inhibited the MAPK pathway in cSCC, and they were potential PDT target genes in cSCC treatment.

## Method

### Data sources

Expressing mRNA profiles and associated clinical data of cSCC were downloaded from the Gene Expression Omnibus (GEO) database (http://www.ncbi.nlm.nih.gov/geo/). Dataset GSE98767, which was performed on GPL10558 and consisted of 15 cSCC patients and three normal subjects with three repetitions (54 samples), was used as the training set to screen different expression genes (DEGs) (18). Dataset GSE45212 performed on GPL570 was used as the validating set, which included 30 cSCC samples and 10 actinic keratosis (AK) samples (40 samples) (19). Dataset GSE84758 was performed on GPL10558 and contained 48 A431 cell samples, among which three control samples, three DT samples, three LC50 PDT-protocol treated samples, and three LC90 PDT-protocol treated samples (12 samples) were used to filter DEGs after PDT treatment (20).

### DEG analysis for normal and tumor groups

The “limma” R package was used to screen DEGs after log2(counts+1) standardizing. The P-value was adjusted by the Bayes test. The adjusted P-value<0.05 and |Log2 (Fold Change) | >1 were set as the cutoff, and the DEGs were applied to further analysis.

### Functional analysis for DEGs

To assess the potential functions of DEGs, the “clusterProfiler” R package was applied to execute Gene Ontology (GO) and Kyoto Encyclopedia of Genes and Genomes (KEGG) analysis. The P-value<0.05 was set as the cutoff criterion.

### Least absolute shrinkage and selection operator regression establish the diagnostic model

The “glmnet” R package and least absolute shrinkage and selection operator regression (Lasso) analysis were used to build the cSCC diagnosis model, thus further filtering cSCC-PDT-related genes. The diagnostic score of each sample was calculated using the following formula: $\;diagnose\;score=\;\sum_{1}^{i}expression\;level\;of\;Gene_{i}\times \beta_{i}$ . The diagnostic score and expression level of selected genes with the maximum AUC value were selected as the boundary values, and the receiver operator characteristic (ROC) curve was drawn in both the training set and validating set.

### Protein–protein interaction network

Protein–protein interaction (PPI) data were obtained from the STRING database (https://cn.string-db.org/, version 11.5) and processed in Cytoscape software.

### Single sample gene set enrichment analysis

The Single sample gene set enrichment analysis (ssGSEA) was applied to explore the enriched scores of MAPK activation level, angiogenesis, cell cycle, differentiation, and proliferation in GSE98767 and GSE84758 using the R package “GSVA” (21). All used GO molecular functional (GOBP) gene sets were acquired from MSigDB (22) (https://www.gsea-msigdb.org/gsea/msigdb/index.jsp, version 7.5.1) and are annotated in **Table S1**.

### Single-cell validation

The CancerSEA database (http://www.biocc.hrbmu.edu.cn/CancerSEA/home.jsp) (23) and EXP0063 were used to validate the correlation between the expression of selected genes and functional states in single-cell lines. EXP0063 (GSE103322) (24) was performed on GPL18573 containing RNA-seq data of 5,902 single cells from 18 head and neck squamous cell carcinoma (HNSCC) patients. The outcome of the tSNE plot and functional states were downloaded from the CancerSEA online tool.

### Cell culture and cell cycle analysis

A431 cells (Thermo Fisher Scientific, USA) were cultured in the 96-well plates at a density of 5 × 10^3^ cells/well and incubated for 24 h. The following compounds were used to treat A431 cells: the EGF group was treated with 100 ng/ml recombinant EGF (sc-4552, Santa Cruz Biotechnology, USA), ERK overexpression group was treated with 10 µM Ceramide C6 (sc-3527, Santa Cruz Biotechnology, USA), and the JNK overexpression group was treated with 500 nM anisomycin (sc-3524, Santa Cruz Biotechnology, USA). Based on our previous research, 400 nmol/L of EtNBSe (Department of Chemistry of Central South University, China) was used to pretreat all groups in the dark for 20 min. Then the cells were irradiated by LED light whose wavelength was 635 nm and power density of 50 mW/cm^2^ for 60 s (2.8 J/cm^2^) (25, 26). After corresponding treatments, cells were harvested and fixed with 75% ethanol overnight at 4°C. The fixed cells were washed three times with PBS and stained darkly with propidium iodide (PI) for 30 min. The stained cells were analyzed by a FACS Calibur flow cytometer (BD Biosciences, USA).

### Statistical analysis

A Spearman analysis was performed to calculate the correlation coefficient between different genes. All statistical analyses were executed in the R language (version 4.1.3) and p<0.05 was considered statistically significant.

## Result

### The 38 cSCC-PDT related genes enriched in the MAPK pathway

**Figure 1:**
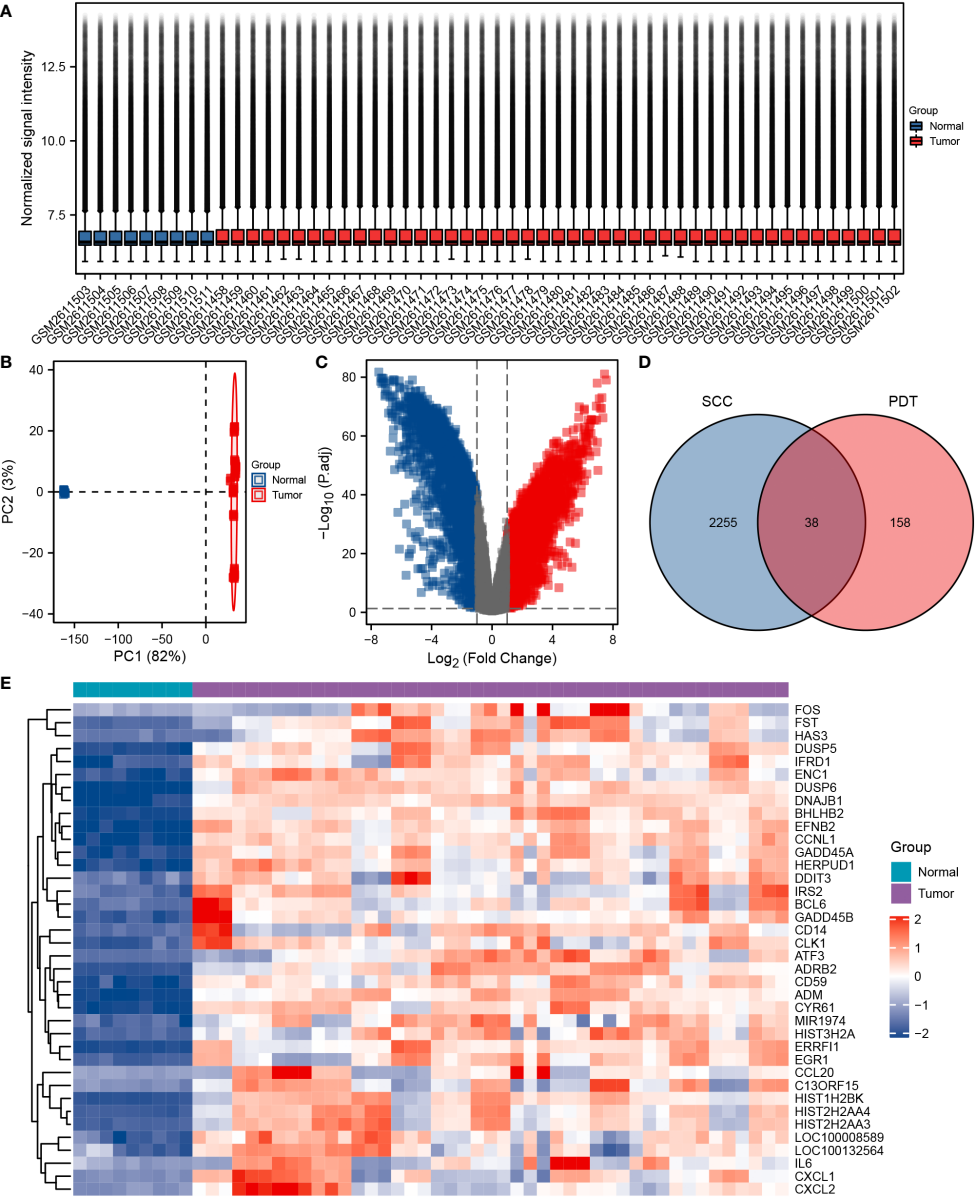
Identification of differentially expressed cSCC-PDT-related genes. **(A)** Box plot of 54 samples of GSE98767 after log_2_ standardization original counts. **(B)** Principal component analysis (PCA) analysis of tumor and a normal group of GSE98767. **(C)** Volcano plot of DEGs in cSCC and normal samples of GSE98767. The red square dots represented upregulated genes, while the green square dots represented downregulated genes (cutoff: adj. P-value<0.05 and |Log_2_ (Fold Change) |>1). **(D)** Venn diagram showing the 38 cSCC-PDT-related genes (the intersection of the cSCC and PDT). **(E)** Heatmap displaying the expression level of 38 cSCC-PDT-related genes in GSE 98767.

The study design is illustrated in **Figure S1**. The nine normal samples and 45 cSCC samples from GSE98676 were used as training sets for differential gene analysis. After Log_2_(counts+1) standardization, the signal density of each sample was generally at the same level for comparison (**Figure 1A**). Principal component analysis (PCA) showed that the two groups had different characteristics (**Figure 1B**). Following the analysis of the GSE98767 dataset using limma, a total of 2,293 DEGs were identified in the cSCC and normal samples, which were displayed in the volcano plot (**Figure 1C**). Dataset GSE84758 was processed in the same way as GSE98767, and 183 DEGs were identified. Then, 38 cSCC-PDT-related genes were obtained from the intersection of the above two DEG sets (**Figure 1D**). **Figure 1E** visually displays the expression level of the 38 genes in GSE98767, while **Table 1** lists information on the 38 genes in detail.

**Table 1:** Detailed data of 38 differential expressed cSCC-PDT-related genes.

Gene.Symbol	GSE98767			GSE84758		
	logFC	P.Value	adj.P.Val	logFC	P.Value	adj.P.Val
HIST1H2BK	4.619604846	3.2167E-17	1.87778E-16	1.31911	6.12E-05	0.009976
ERRFI1	4.277016012	1.41405E-20	9.2757E-20	2.833846	1.29E-08	1.88E-05
DUSP6	4.032873036	8.16355E-30	7.60036E-29	3.046067	2.39E-09	6.9E-06
ADM	3.963986533	1.00703E-24	7.68616E-24	1.135633	2.02E-06	0.000777
FST	3.714380164	1.49909E-10	6.73409E-10	1.602592	6.56E-07	0.000345
BHLHB2	3.660332346	2.41494E-22	1.69131E-21	1.779734	1.77E-06	0.000722
CYR61	3.460940803	4.58412E-23	3.29964E-22	3.036142	5.96E-09	1.22E-05
ADRB2	3.044026009	2.97859E-16	1.67296E-15	2.437377	1.65E-07	0.000114
DUSP5	2.847290007	5.34772E-20	3.44196E-19	1.460276	1.57E-06	0.000673
HIST2H2AA4	2.724052988	1.01847E-13	5.18591E-13	1.200566	7.65E-07	0.00039
EFNB2	2.709520902	2.83635E-28	2.49632E-27	1.215793	3.36E-05	0.006438
HIST2H2AA3	2.675518726	4.78866E-12	2.2851E-11	1.228328	4.5E-06	0.001351
DNAJB1	2.57353698	4.89735E-39	7.0148E-38	1.344019	9.32E-06	0.002269
EGR1	2.560813139	2.52611E-12	1.21651E-11	3.634255	7.14E-10	3.83E-06
GADD45A	2.44572528	4.25018E-19	2.64854E-18	1.09097	5.53E-06	0.001573
CXCL1	2.252951442	1.28374E-05	4.36089E-05	2.16827	1.07E-07	9.02E-05
HAS3	2.187433524	4.91931E-08	1.94021E-07	1.039821	5.13E-05	0.008674
HERPUD1	2.134388824	1.48123E-20	9.71393E-20	1.002377	9.02E-06	0.002235
CCNL1	2.060797924	6.12652E-28	5.31988E-27	1.531865	2.08E-06	0.000777
MIR1974	2.053170518	1.13558E-10	5.13531E-10	2.579296	0.000258	0.025853
LOC100008589	1.967748936	7.25901E-12	3.44471E-11	1.843204	6.63E-07	0.000345
CCL20	1.794241775	0.000524911	0.001547247	1.158155	0.000195	0.021842
LOC100132564	1.732251157	3.18461E-07	1.19963E-06	1.95103	1.38E-07	0.000104
CD14	1.700324014	1.14268E-09	4.89735E-09	1.11706	7.12E-06	0.001878
ENC1	1.653764421	7.67012E-18	4.58172E-17	1.129762	0.000124	0.016402
CD59	1.586550226	3.87304E-23	2.79251E-22	1.081346	9.92E-05	0.01401
ATF3	1.475421346	4.40731E-09	1.83767E-08	4.302127	5.42E-10	3.76E-06
IFRD1	1.386579824	2.63634E-15	1.42823E-14	1.122456	1.43E-06	0.000627
IRS2	1.354135019	1.92067E-09	8.1541E-09	1.922806	4.6E-06	0.001363
BCL6	1.340384462	6.01622E-09	2.49152E-08	1.873812	4.7E-07	0.000263
GADD45B	1.263438308	5.43368E-14	2.79334E-13	2.55823	3E-08	2.98E-05
C13ORF15	1.258333669	2.41386E-05	8.03098E-05	2.651124	9.9E-08	8.61E-05
HIST3H2A	1.198692676	3.1811E-11	1.47308E-10	1.090508	5.43E-05	0.008964
IL6	1.193940996	0.000902549	0.00259803	1.953634	1.61E-06	0.000681
FOS	1.148625326	0.002655586	0.007273109	7.523147	6.92E-11	2.25E-06
CXCL2	1.13684321	2.58403E-05	8.5747E-05	3.846698	5.33E-08	5E-05
DDIT3	1.060898997	9.09349E-08	3.52906E-07	3.373708	3.22E-09	8.59E-06
CLK1	1.016256653	6.66236E-13	3.27574E-12	1.235296	3.43E-05	0.006521

**Figure 2:**
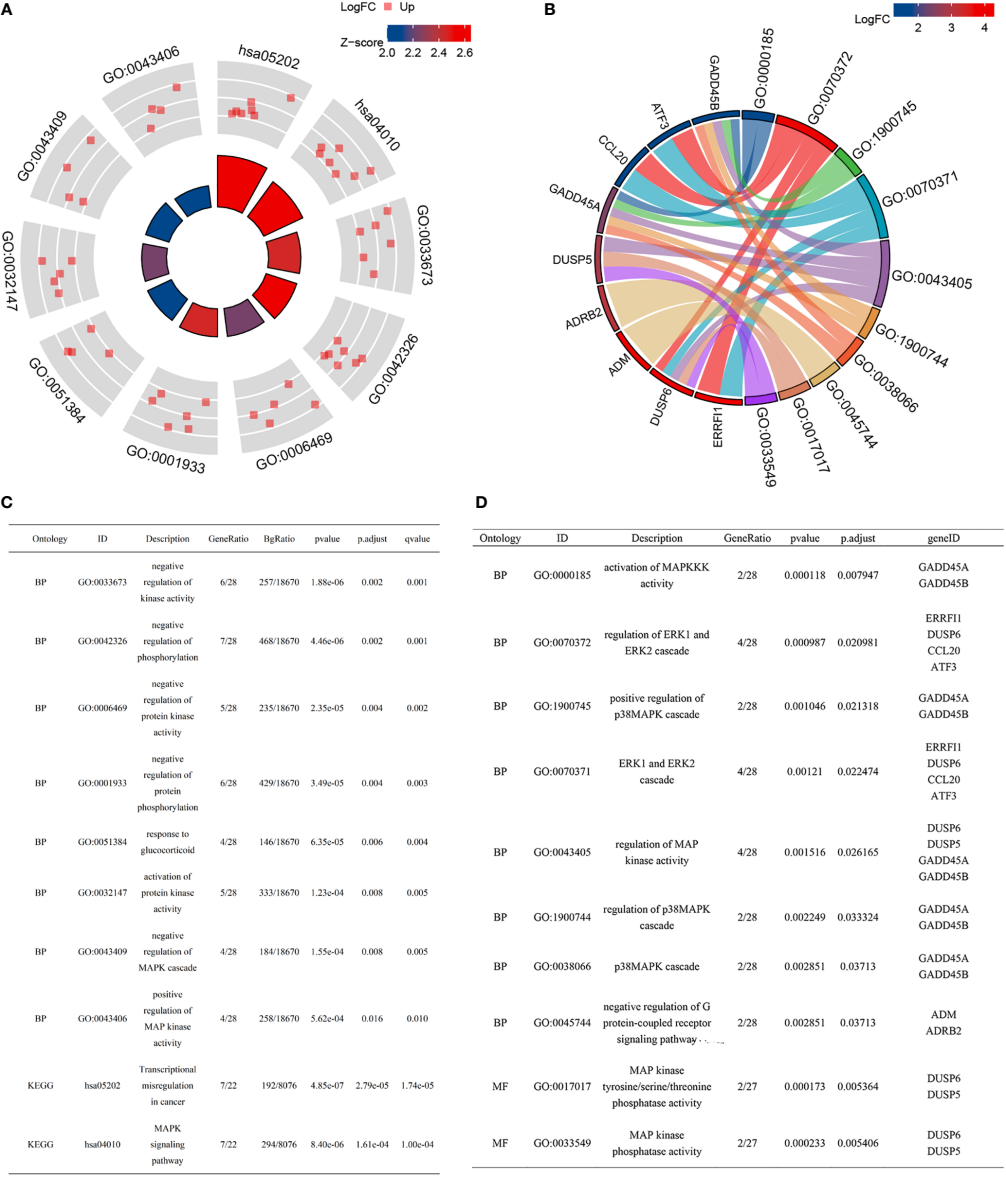
GO and KEGG enrichment analysis of 38 cSCC-PDT-related genes. **(A)** Radar plot of GO and KEGG analysis. **(B)** A chord gram showing the subordination between genes and the GO terms. **(C)** The list elaborates on the results of GO and KEGG analysis. **(D)** GO terms relevant to the MAPK pathway are highlighted.

Subsequently, we conducted a functional enrichment analysis for these 38 genes. GO analysis included 28 cSCC-PDT-related genes, which were mainly enriched in biological processes (BP), such as negative regulation of kinase activity, negative regulation of phosphorylation, and negative regulation of protein kinase activity (**Figure 2C**). Interestingly, DUSP6 participated in five different GO pathways (**Figure 2B**). KEGG analysis included 22 cSCC-PDT-related genes, which were significantly enriched in the MAPK signaling pathway (**Figures 2A–C**). To further evaluate the regulation of cSCC-PDT-related genes on the MAPK pathway, the results relevant to MAPK of GO analysis are highlighted in **Figure 2D**, indicating that cSCC-PDT-related genes are engaged in the MAPK pathway.

### Establishment and validation of diagnosis model

The Lasso regression was applied to establish a cSCC diagnostic model to further select key cSCC-PDT-related genes in GSE98767. Eventually, four of the 38 cSCC-PDT-related genes were included in the diagnostic model (**Figures S2A, B**). The ROC curve was used to assess the diagnostic utility of the diagnostic score and the expression level of DUSP6, EFNB2, DNAJB1, and CCNL1 in GSE98767 (cSCC and normal) and GSE45216 (cSCC and AK), and the boundary value with maximal ACU was selected as the cutoff (**Figures S2C–F**). In the training set, the diagnostic model had remarkable diagnostic utility (AUC = 1.000), while in the validation set, the overall diagnostic utility of the model was also noteworthy (AUC = 0.760).

### DUSP6, EFNB2, DNAJB1, and CCNL1 were correlated with MAPK and cell cycle

**Figure 3:**
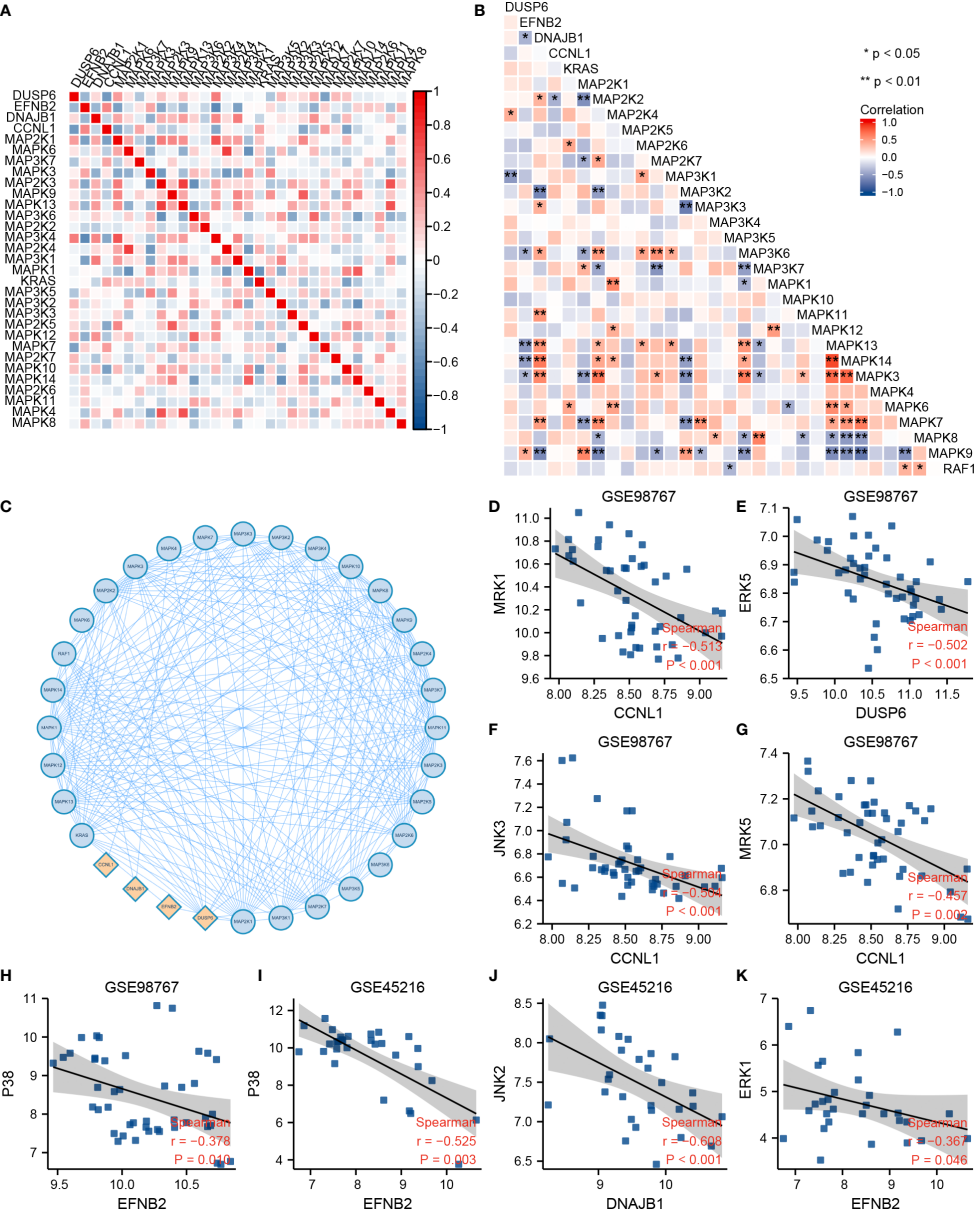
Correlation analysis between DUSP6, EFNB2, DNAJB1, CCNL1, and MAPK pathway. **(A)** A heatmap of correlative coefficients between four cSCC-PDT genes and significant MAPK genes in GSE98767. **(B)** A heatmap of correlative coefficients between four cSCC-PDT genes and significant MAPK genes in GSE45216. **(C)** The PPI network of four cSCC-PDT genes and significant MAPK genes. (**D–K**) Spearman analysis of four cSCC-PDT genes and MAPK pathway promoter.

The correlations between DUSP6, EFNB2, DNAJB1, CCNL1, and GOBP MAPK cascade gene sets were calculated by Spearman analysis. **Figures 3A, B** display a correlative coefficient between four cSCC-PDT genes and significant MAPK genes of the GOBP_MAPK_cascade in GSE98767 (**Figure 3A**) and GSE45216 (**Figure 3B**). EFNB2 and DNAJB1 were reversely correlated with MAP3K6/MEKK6, MAPK3/ERK1, MAPK13/p38δ, and MAPK14/p38α. The PPI network exhibited the protein interactions of the above genes (**Figure 3C**). We then estimated the correlation between the four cSCC-PDT genes and the MAPK pathway promoter. The expression level of four cSCC-PDT genes was negatively correlated with some general MAPK pathway promoters, MRK, ERK, JNK, and p38 (11) in cSCC samples (**Figures 3D–K**).

**Figure 4:**
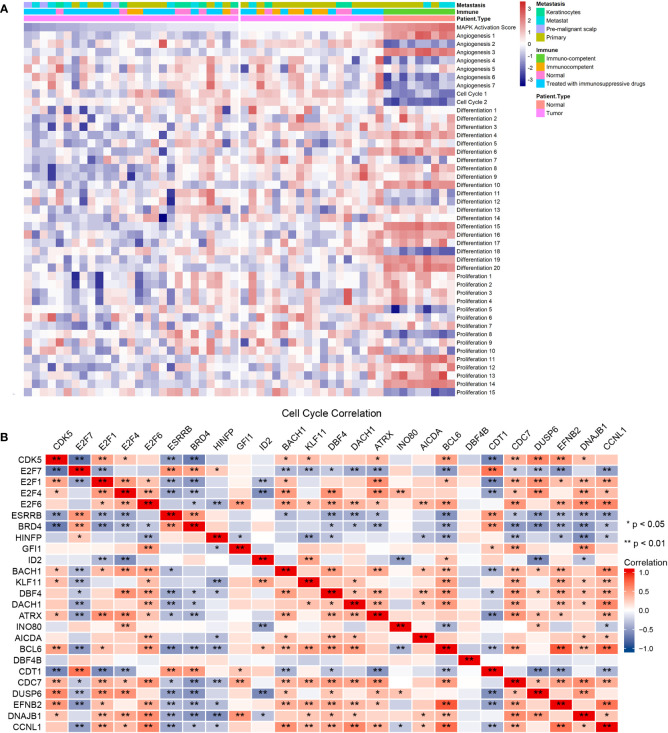
The four cSCC-PDT-related genes were correlated with the cell cycle gene. **(A)** The result of ssGSEA analysis of GSE98767 sorted by MAPK activation level. **(B)** Correlation coefficient matrix of four cSCC-PDT-related genes and cell cycle genes.

Previous studies have indicated that MAPK activation stimulates cell cycle transitions (14, 15). Here, we conducted ssGSEA on the cSCC sample of GES98767 to estimate the relationship between MAPK activation level and cell cycle. The normal group had a higher MAPK activation score, differentiation-enriched score, and lower cell cycle enriched score, angiogenesis-enriched score than the cSCC group (**Figure 4A**). Different MAPK promoters induce completely different functions, as JNK and p38 mediate cell apoptosis and downregulate in many tumors (27) whereas hyperactivation of ERK plays a crucial role in tumor proliferation (28). We wonder if the greater decline in JNK and p38 contributes to a lower MAPK activation score in cSCC. Consequently, the correlation between four cSCC-PDT-related genes and cell cycle genes was computed by spearman analysis in which DUSP6, EFNB2, DNAJB1, and CCNL1 were significantly correlated with significant cell cycle genes of GOBP_cell_cycle_process (**Figure 4B**). To explore how these genes regulate the MAPK signal pathway, we searched relevant signal maps from the KEGG database (https://www.genome.jp/kegg/), where DUSP6 inhibited ERK through dephosphorylation, thus inhibiting the EGF-EGFR-Ras-ERK signal pathway and preventing cell proliferation and differentiation (**Figure S3** **)**.

### The four SCC-PDT related genes were potential PDT targets regulating the cell cycle by adjusting MAPK

**Figure 5:**
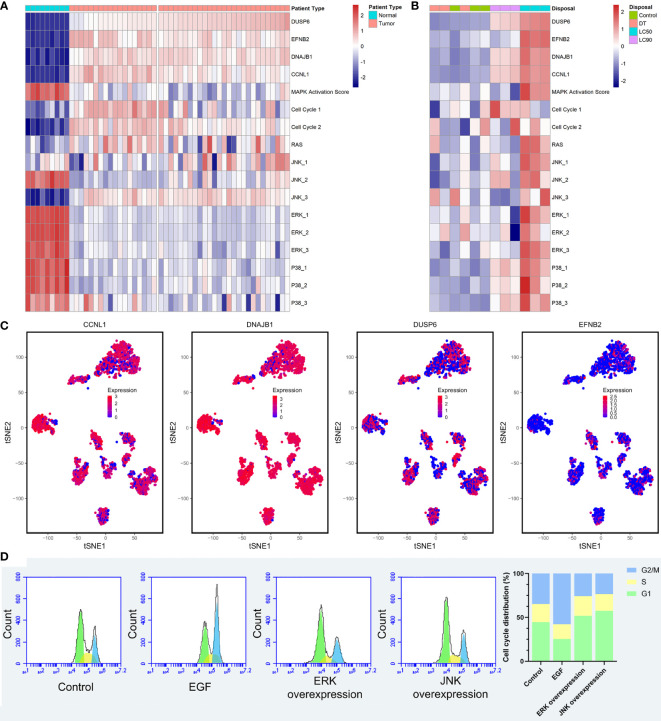
Validation of the relationship among the four SCC-PDT related genes, the MAPK pathway, and the cell cycle. **(A)** A heatmap of ssGESA analysis on GSE98767, which is ordered by the expression level of DUSP6. **(B)** A heatmap of ssGESA analysis on GSE84758, which is ordered by the expression level of DUSP6. The treatment of A431 cells was annotated at the top. **(C)** The tSNA plot displayed the expression level of CCNL1, DNAJB1, DUSP6, and EFNB2. **(D)** PI staining and flow cytometry were performed to estimate the A431 cell cycle phase distribution. Recombinant EGF sc-4552 (100 ng/ml), ERK activator Ceramide C6 (10 µM), and JNK activator anisomycin (500 µM) were used to treat A431 cells before PDT treatment (2.8 J/cm^2^, 635 nm). The bar chart shows the composition ratio of cells in the G1 phase, S phase, and G2/M phase.

To verify the relationship among the four SCC-PDT related genes, MAPK pathways, and cell cycle, we distinguished RAS, JNK, ERK, and P38 pathways in ssGESA analysis. The pathway annotation is shown in **Table S1**. At the pathway level of GSE98767, SCC samples had higher activity in the cell cycle, JNK_3 (negative regulation of JNK) than normal cells, while MAPK Activation Score, ERK and p38 were lower (**Figure 5A**). In the PDT-processed dataset, GSE84758, LC50 PDT-protocol treatment significantly increased the activity of MAPK Activation Score, RAS, JNK, ERK, and p38 (JNK, ERK, and p38 pathways included positive and negative regulatory pathways), as the LC90 PDT-protocol treatment reduced it (**Figure 5B**). The sample size of GSE84758 was too small to give a definitive inference, but enough to indicate some interesting phenomena.

After that, we validated the above relationship at the single-cell level. The expression level of four cSCC-PDT-related genes in 5,902 HNSCC single cells of GSE103322 was displayed in the tSNE plot, and CCNL1 and DNAJB1 were highly expressed in most of the cells (**Figure 5C**). The gene expression level and cell cycle function state were comprehensively ranked, and the top five single cells with high cell cycle function states are shown in the radar plot (**Figure S4**). To elucidate the effect of the MAPK pathway on cell cycles after PDT, A431 cells were incubated with 100 ng/ml recombinant EGF, 10 µM Ceramide C6 as the ERK overexpression group, and 500 nM anisomycin as the JNK overexpression group, respectively, before PDT treatment. The number of cells in each cell cycle was counted by flow cytometry. The results showed that more cells stagnated in the G1 phase in the JNK overexpression group compared to the control group **(** **Figure 5D** **)**. Incidentally, ERK overexpression was supposed to promote cell proliferation and differentiation, producing effects similar to those of EGF treatment (29, 30). In this study, A431 cells with ERK overexpression performed similarly to cells with JNK overexpression after PDT treatment, resulting in inhibition of the cell cycle. In fact, different types of photosensitizers and cell lines activated MAPK pathways various. As EtNBSe is a newly developed photosensitizer, more pieces of evidence are needed to explore this phenomenon and its mechanism.

## Discussion

The ascending incidence rates of cSCC increase the healthcare burden (31). PDT is recommended for treating cSCC because of its high tissue selectivity, good cosmetic outcome, comparatively less invasiveness, low recurrence rate, and, especially, safety for patients who cannot tolerate surgery. As for treating invasive cSCC, although many studies have demonstrated the efficacy of PDT (3, 32, 33), it has not been regarded as the first-line solution in the European guidelines (2). PDT still requires some modifications to be effectively used for invasive cSCC due to its inability to reach an adequate deep layer of skin. The combination of PDT and adjuvant therapy might expand the clinical application of PDT. Mechanism-based studies can provide evidence for the security and effectiveness of this combination. Previous studies illustrated that the MAPK pathway regulated cell proliferation, invasion, and differentiation in cSCC. PDT was proved to suppress the ERK pathway and activate the JNK pathway, and inhibit cell cycles. Nonetheless, there are rare reports about the comprehensive mechanism of PDT for cSCC treatment. Here we explored whether PDT inhibition of cSCC proliferation was related to MAPK signaling and its regulation of the G1 to G2/M phase.

In our study, we found 38 cSCC-PDT-related genes through differential analysis and intersection of differential gene sets. Then, we conducted GO/KEGG functional enrichment analysis for these 38 genes. The results indicated that most cSCC-PDT-related genes are enriched in BP, such as negative regulation of kinase activity, phosphorylation, and protein kinase activity. Many cSCC-PDT-related genes engaged in the MAPK pathway, which was consistent with previous studies (20, 34). Activation and inhibition of the MAPK pathway in cSCC are mainly affected by two aspects. On the one hand, the Ras/ERK signaling which is correlated to cancer initiation and development is upregulated in cSCC. Pierceall et al. found activating mutations and amplification of the Ras oncogene, which was an upstream activator of the Raf/Mek/Erk1/Erk2 kinase pathway, a typical MAPK pathway, in cSCC (35). Subsequent research determined that activating mutations in Ras could promote cSCC formation (36). Ratushny et al. clarified that MEK/ERK was one of the pro-oncogenic signaling pathways (37). On the other hand, JNK/p38 is downregulated so that the apoptosis of tumor cells is inhibited. Previous studies also illustrated these results (12, 16). In our study, MAPK activation was inhibited in tumor cells, probably because the downregulation of JNK was greater than the upregulation of ERK. As for the effect of PDT on the MAPK pathway, recent studies have reported that ZnPc-PDT (zinc phthalocyanine-PDT) could downregulate the expression and activity of MAPK (38) and PDT downregulated MAPK1 and MAPK3 (20). The above results indicated that the upregulation of the MAPK pathway might induce the occurrence and progression of cSCC, and PDT could suppress MAPK signaling and exert therapeutic effects. Additionally, it suggested that cSCC-PDT-related genes are probably engaged in the MAPK pathway. Previous research indicated that M-PDT could restrain MAPK activation by activating JNK (16). Furthermore, our flow cytometry results revealed that the mechanism of PDT inhibiting the proliferation of cSCC was that the upregulated JNK blocked the cell cycle transition from G1 to G2/M.

Next, we applied Lasso regression and established a diagnostic model based on four key cSCC-PDT-related genes, including DUSP6, EFNB2, DNAJB1, and CCNL1. After that, we preliminarily verified that the expression levels of four cSCC-PDT-related genes were negatively correlative with some MAPK pathway promoters by Spearman analysis. By ssGSEA analysis, we found that high MAPK activation was related to cell cycle transition. DUSP6 is a kind of dual-specificity mitogen-activated protein kinase phosphatase (MKP or DUSP), which is an established negative regulator of the MAPK pathway in mammalian cells (39). Groom et al. determined that DUSP6 preferentially inhibits ERK (40) and that the reduction of DUSP6 caused by ROS was correlated with high ERK activity (41). Additionally, the heat map showed high expression of DUSP6 in tumor cells compared with normal cells in **Figure 2E**, because DUSP6 was upregulated in response to elevated ERK signaling in cSCC with Ras activating mutation, where it was speculated to restrain ERK signaling (42, 43). Kim et al. found that EFNB2 inhibited vascular endothelial growth factor (VEGF)- and angiopoietin-1 (Ang1)-induced Ras/MAPK activities (44). In their study, Muller et al. observed a high prevalence of CCNL1 gene overexpression in HNSCCs and indicated that CCNL1 could be a regulator of cell-cycle transition (45). The function of DNAJB1 has rarely been reported. Furthermore, the single-cell analysis in our study also proved that CCNL1 and DNAJB1 were highly expressed in HNSCC.

Taken together, we speculated that increasing four cSCC-PDT-related genes inhibited the MAPK pathway in cSCC. Concretely, DUSP6 probably inhibited ERK5 MAPK signaling and EFNB2 restrained ERK1/2 and p38 MAPK signaling. DNAJB1 and CCNL1 were potential genes that repress JNK MAPK signaling. We innovatively established a connection between PDT, MAPK, and the cell cycle through bioinformatics analysis, proposing that PDT could repress the G1 to G2/M phase and inhibit tumor growth by regulating the MAPK pathway in cSCC. The results of flow cytometry were consistent with the abovementioned findings. There are still inescapable limitations to our research. Though we have screened for cSCC-PDT-related genes with bioinformatics analysis, attempting to clarify the roles of the MAPK pathway for treating PDT for cSCC, more *in vivo* and *in vitro* experiments are needed for the direct biological evidence to elucidate the exact mechanism of action. Meanwhile, we found a potential therapeutic target of PDT, which may be related to MAPK activation and cell cycle alternation. However, the definite effect of these four genes on cell cycles requires more exploration to expound.

## Conclusion

CCNL1, DNAJB1, DUSP6, and EFNB2 were upregulated in cSCC or LC50 PDT-protocol treated A431 cells, which could be used as diagnostic markers for cSCC. Based on our results, we speculated that increased CCNL1, DNAJB1, DUSP6, and EFNB2 inhibited the MAPK pathway in cSCC. Despite a lower MAPK activation score, SCC appeared to have higher activity in the cell cycle. Strikingly, LC50 PDT-protocol treated A431 cell showed high levels of CCNL1, DNAJB1, DUSP6, and EFNB2 and extensive MAPK activation. Generally, we innovatively found a connection between PDT, MAPK and the cell cycle *via* CCNL1, DNAJB1, DUSP6, and EFNB2. *In vitro*, we discovered that PDT treatment suppressed the G1 to G2/M phase in JNK overexpressed A431 cells. In other words, CCNL1, DNAJB1, DUSP6, and EFNB2 are potential PDT target genes in cSCC treatment.

## Data availability statement

The original contributions presented in the study are included in the article/**Supplementary Material**. Further inquiries can be directed to the corresponding authors.

## Author contributions

YZ and JD wrote the first draft of the manuscript. YL, HW, and DZ contributed to conception and design of the study. YZ, JD, and YL contributed equally to this work. All authors contributed to the article and approved the submitted version.

## Funding

This work is supported by the Hunan Medical Technology Innovation Guidance Fund grant no. 2020SK53615, the Natural Science Foundation of Hunan Province grant no. 2020JJ8015, 2019JJ40468, and 2019JJ80031, and the Changsha Natural Science Foundation grant no. kq2001041.

## Conflict of interest

The authors declare that the research was conducted in the absence of any commercial or financial relationships that could be construed as a potential conflict of interest.

## Publisher’s note

All claims expressed in this article are solely those of the authors and do not necessarily represent those of their affiliated organizations, or those of the publisher, the editors and the reviewers. Any product that may be evaluated in this article, or claim that may be made by its manufacturer, is not guaranteed or endorsed by the publisher.

## Supplementary material

Supplementary Figure 1 The study design identified 4 potential targeted genes after PDT treatment regulating MAPK activation and cell cycle in cSCC.

Supplementary Figure 2 Establishment and validation of diagnosis model by Lasso regression and ROC curve. **(A)** Coefficient profiles of 38 cSCC-PDT-related genes. **(B)** Selection of the optimal lambda in the Lasso model. **(C–F)** ROC curve of diagnosis model and expression level of above 4 genes.

Supplementary Figure 3 KEGG pathway map of MAPK signaling pathway (hsa04010, https://www.genome.jp/pathway/hsa04010+1848)

Supplementary Figure 4 Radar plot of top five single cells with high cell cycle function state after comprehensively ranked by gene expression level and cell cycle function state. **(A–D)** The single cell set was sequentially sorted by expression levels of CCNL1, DNAJB1, EFNB2 and DUSP6.

Supplementary Table 1 GO terms annotation of ssGSEA analysis.

## References

[1] Global Burden of Disease Cancer C Fitzmaurice C Abate D Abbasi N Abbastabar H Abd-Allah F. Global, regional, and national cancer incidence, mortality, years of life lost, years lived with disability, and disability-adjusted life-years for 29 cancer groups, 1990 to 2017: A systematic analysis for the global burden of disease study. JAMA Oncol (2019) 5(12):1749–68. doi: 10.1001/jamaoncol.2019.2996 PMC6777271 31560378

[2] Stratigos AJ Garbe C Dessinioti C Lebbe C Bataille V Bastholt L. European Interdisciplinary guideline on invasive squamous cell carcinoma of the skin: Part 2. Treat Eur J Cancer (2020) 128:83–102. doi: 10.1016/j.ejca.2020.01.008 32113942

[3] Keyal U Bhatta AK Zhang G Wang XL. Present and future perspectives of photodynamic therapy for cutaneous squamous cell carcinoma. J Am Acad Dermatol (2019) 80(3):765–73. doi: 10.1016/j.jaad.2018.10.042 30393093

[4] Kwiatkowski S Knap B Przystupski D Saczko J Kedzierska E Knap-Czop K. Photodynamic therapy - mechanisms, photosensitizers and combinations. BioMed Pharmacother (2018) 106:1098–107. doi: 10.1016/j.biopha.2018.07.049 30119176

[5] Donohoe C Senge MO Arnaut LG Gomes-da-Silva LC. Cell death in photodynamic therapy: From oxidative stress to anti-tumor immunity. Biochim Biophys Acta Rev Cancer (2019) 1872(2):188308. doi: 10.1016/j.bbcan.2019.07.003 31401103

[6] Brash DE Rudolph JA Simon JA Lin A McKenna GJ Baden HP. A role for sunlight in skin cancer: Uv-induced P53 mutations in squamous cell carcinoma. Proc Natl Acad Sci U.S.A. (1991) 88(22):10124–8. doi: 10.1073/pnas.88.22.10124 PMC52880 1946433

[7] Ziegler A Jonason AS Leffell DJ Simon JA Sharma HW Kimmelman J. Sunburn and P53 in the onset of skin cancer. Nature (1994) 372(6508):773–6. doi: 10.1038/372773a0 7997263

[8] South AP Purdie KJ Watt SA Haldenby S den Breems N Dimon M. Notch1 mutations occur early during cutaneous squamous cell carcinogenesis. J Invest Dermatol (2014) 134(10):2630–8. doi: 10.1038/jid.2014.154 PMC4753672 24662767

[9] Simanshu DK Nissley DV McCormick F. Ras proteins and their regulators in human disease. Cell (2017) 170(1):17–33. doi: 10.1016/j.cell.2017.06.009 28666118 PMC5555610

[10] Corchado-Cobos R Garcia-Sancha N Gonzalez-Sarmiento R Perez-Losada J Canueto J. Cutaneous squamous cell carcinoma: From biology to therapy. Int J Mol Sci (2020) 21(8):2956. doi: 10.3390/ijms21082956 PMC7216042 32331425

[11] Cargnello M Roux PP. Activation and function of the mapks and their substrates, the mapk-activated protein kinases. Microbiol Mol Biol Rev (2011) 75(1):50–83. doi: 10.1128/MMBR.00031-10 21372320 PMC3063353

[12] Vin H Ojeda SS Ching G Leung ML Chitsazzadeh V Dwyer DW. Braf inhibitors suppress apoptosis through off-target inhibition of jnk signaling. Elife (2013) 2:e00969. doi: 10.7554/eLife.00969 24192036 PMC3814616

[13] Que SKT Zwald FO Schmults CD. Cutaneous squamous cell carcinoma: Incidence, risk factors, diagnosis, and staging. J Am Acad Dermatol (2018) 78(2):237–47. doi: 10.1016/j.jaad.2017.08.059 29332704

[14] Reddy D Kumavath R Ghosh P Barh D. Lanatoside c induces G2/M cell cycle arrest and suppresses cancer cell growth by attenuating mapk, wnt, jak-stat, and Pi3k/Akt/Mtor signaling pathways. Biomolecules (2019) 9(12):792. doi: 10.3390/biom9120792 PMC6995510 31783627

[15] Whitaker RH Cook JG. Stress relief techniques: P38 mapk determines the balance of cell cycle and apoptosis pathways. Biomolecules (2021) 11(10):1444. doi: 10.3390/biom11101444 34680077 PMC8533283

[16] Liu J Yan G Chen Q Zeng Q Wang X. Modified 5-aminolevulinic acid photodynamic therapy (M-pdt) inhibits cutaneous squamous cell carcinoma cell proliferation *Via* targeting Pp2a/Pp5-mediated mapk signaling pathway. Int J Biochem Cell Biol (2021) 137:106036. doi: 10.1016/j.biocel.2021.106036 34217813

[17] Tan P Cai H Wei Q Tang X Zhang Q Kopytynski M. Enhanced chemo-photodynamic therapy of an enzyme-responsive prodrug in bladder cancer patient-derived xenograft models. Biomaterials (2021) 277:121061. doi: 10.1016/j.biomaterials.2021.121061 34508957

[18] Inman GJ Wang J Nagano A Alexandrov LB Purdie KJ Taylor RG. The genomic landscape of cutaneous scc reveals drivers and a novel azathioprine associated mutational signature. Nat Commun (2018) 9(1):3667. doi: 10.1038/s41467-018-06027-1 30202019 PMC6131170

[19] Lambert SR Mladkova N Gulati A Hamoudi R Purdie K Cerio R. Key differences identified between actinic keratosis and cutaneous squamous cell carcinoma by transcriptome profiling. Brit J Cancer (2014) 110(2):520–9. doi: 10.1038/bjc.2013.760 PMC3899778 24335922

[20] Weijer R Clavier S Zaal EA Pijls MM van Kooten RT Vermaas K. Multi-omic profiling of survival and metabolic signaling networks in cells subjected to photodynamic therapy. Cell Mol Life Sci (2017) 74(6):1133–51. doi: 10.1007/s00018-016-2401-0 PMC5309296 27803950

[21] Hanzelmann S Castelo R Guinney J. Gsva: Gene set variation analysis for microarray and rna-seq data. BMC Bioinf (2013) 14:7. doi: 10.1186/1471-2105-14-7 PMC3618321 23323831

[22] Liberzon A Subramanian A Pinchback R Thorvaldsdottir H Tamayo P Mesirov JP. Molecular signatures database (Msigdb) 3.0. Bioinformatics (2011) 27(12):1739–40. doi: 10.1093/bioinformatics/btr260 PMC3106198 21546393

[23] Yuan H Yan M Zhang G Liu W Deng C Liao G. Cancersea: A cancer single-cell state atlas. Nucleic Acids Res (2019) 47(D1):D900–D8. doi: 10.1093/nar/gky939 PMC6324047 30329142

[24] Puram SV Tirosh I Parikh AS Patel AP Yizhak K Gillespie S. Single-cell transcriptomic analysis of primary and metastatic tumor ecosystems in head and neck cancer. Cell (2017) 171(7):1611–24 e24. doi: 10.1016/j.cell.2017.10.044 29198524 PMC5878932

[25] Liu A Zhang W Chen Y Zhou D Wang Z Kang J. Etnbse-pdt inhibited proliferation and induced autophagy of hne-1 cells *Via* downregulating the Wnt/Beta-catenin signaling pathway. Photodiagnosis Photodyn Ther (2019) 26:65–72. doi: 10.1016/j.pdpdt.2019.02.024 30831261

[26] Chen J Zhou D Kang J Liu C Huang R Jiang Z. Er stress modulates apoptosis in A431 cell subjected to etnbse-pdt *Via* the perk pathway. Photodiagnosis Photodyn Ther (2021) 34:102305. doi: 10.1016/j.pdpdt.2021.102305 33901688

[27] Yue J Lopez JM. Understanding mapk signaling pathways in apoptosis. Int J Mol Sci (2020) 21(7):2346. doi: 10.3390/ijms21072346 PMC7177758 32231094

[28] Guo YJ Pan WW Liu SB Shen ZF Xu Y Hu LL. Erk/Mapk signalling pathway and tumorigenesis. Exp Ther Med (2020) 19(3):1997–2007. doi: 10.3892/etm.2020.8454 32104259 PMC7027163

[29] Gilaberte Y Milla L Salazar N Vera-Alvarez J Kourani O Damian A. Cellular intrinsic factors involved in the resistance of squamous cell carcinoma to photodynamic therapy. J Invest Dermatol (2014) 134(9):2428–37. doi: 10.1038/jid.2014.178 24717244

[30] Olsen CE Weyergang A Edwards VT Berg K Brech A Weisheit S. Development of resistance to photodynamic therapy (Pdt) in human breast cancer cells is photosensitizer-dependent: Possible mechanisms and approaches for overcoming pdt-resistance. Biochem Pharmacol (2017) 144:63–77. doi: 10.1016/j.bcp.2017.08.002 28784290

[31] Tokez S Wakkee M Louwman M Noels E Nijsten T Hollestein L. Assessment of cutaneous squamous cell carcinoma (Cscc) in situ incidence and the risk of developing invasive cscc in patients with prior cscc in situ vs the general population in the Netherlands, 1989-2017. JAMA Dermatol (2020) 156(9):973–81. doi: 10.1001/jamadermatol.2020.1988 PMC7330830 32609322

[32] de Albuquerque IO Nunes J Figueiro Longo JP Muehlmann LA Azevedo RB. Photodynamic therapy in superficial basal cell carcinoma treatment. Photodiagnosis Photodyn Ther (2019) 27:428–32. doi: 10.1016/j.pdpdt.2019.07.017 31349099

[33] Tampa M Sarbu MI Matei C Mitran CI Mitran MI Caruntu C. Photodynamic therapy: A hot topic in dermato-oncology. Oncol Lett (2019) 17(5):4085–93. doi: 10.3892/ol.2019.9939 PMC6444307 30944601

[34] Allegra A Pioggia G Tonacci A Musolino C Gangemi S. Oxidative stress and photodynamic therapy of skin cancers: Mechanisms, challenges and promising developments. Antioxidants (Basel) (2020) 9(5):448. doi: 10.3390/antiox9050448 PMC7278813 32455998

[35] Pierceall WE Goldberg LH Tainsky MA Mukhopadhyay T Ananthaswamy HN. Ras gene mutation and amplification in human nonmelanoma skin cancers. Mol Carcinog (1991) 4(3):196–202. doi: 10.1002/mc.2940040306 2064725

[36] Khavari PA. Modelling cancer in human skin tissue. Nat Rev Cancer (2006) 6(4):270–80. doi: 10.1038/nrc1838 16541145

[37] Ratushny V Gober MD Hick R Ridky TW Seykora JT. From keratinocyte to cancer: The pathogenesis and modeling of cutaneous squamous cell carcinoma. J Clin Invest (2012) 122(2):464–72. doi: 10.1172/JCI57415 PMC3266779 22293185

[38] Ogbodu RO Nitzsche B Ma A Atilla D Gurek AG Hopfner M. Photodynamic therapy of hepatocellular carcinoma using tetra-triethyleneoxysulfonyl zinc phthalocyanine as photosensitizer. J Photochem Photobiol B (2020) 208:111915. doi: 10.1016/j.jphotobiol.2020.111915 32480203

[39] Kidger AM Keyse SM. The regulation of oncogenic Ras/Erk signalling by dual-specificity mitogen activated protein kinase phosphatases (Mkps). Semin Cell Dev Biol (2016) 50:125–32. doi: 10.1016/j.semcdb.2016.01.009 PMC5056954 26791049

[40] Groom LA Sneddon AA Alessi DR Dowd S Keyse SM. Differential regulation of the map, sap and Rk/P38 kinases by Pyst1, a novel cytosolic dual-specificity phosphatase. EMBO J (1996) 15(14):3621–32. doi: 10.1002/j.1460-2075.1996.tb00731.x PMC451978 8670865

[41] Chan DW Liu VW Tsao GS Yao KM Furukawa T Chan KK. Loss of Mkp3 mediated by oxidative stress enhances tumorigenicity and chemoresistance of ovarian cancer cells. Carcinogenesis (2008) 29(9):1742–50. doi: 10.1093/carcin/bgn167 18632752

[42] Packer LM East P Reis-Filho JS Marais R. Identification of direct transcriptional targets of (V600e)Braf/Mek signalling in melanoma. Pigment Cell Melanoma Res (2009) 22(6):785–98. doi: 10.1111/j.1755-148X.2009.00618.x 19682280

[43] Vartanian S Bentley C Brauer MJ Li L Shirasawa S Sasazuki T. Identification of mutant K-Ras-Dependent phenotypes using a panel of isogenic cell lines. J Biol Chem (2013) 288(4):2403–13. doi: 10.1074/jbc.M112.394130 PMC3554910 23188824

[44] Kim I Ryu YS Kwak HJ Ahn SY Oh JL Yancopoulos GD. Ephb ligand, Ephrinb2, suppresses the vegf- and angiopoietin 1-induced Ras/Mitogen-activated protein kinase pathway in venous endothelial cells. FASEB J (2002) 16(9):1126–8. doi: 10.1096/fj.01-0805fje 12039842

[45] Muller D Millon R Theobald S Hussenet T Wasylyk B du Manoir S. Cyclin L1 (Ccnl1) gene alterations in human head and neck squamous cell carcinoma. Br J Cancer (2006) 94(7):1041–4. doi: 10.1038/sj.bjc.6603036 PMC2361229 16598186

