# Supplementary figures and images for: Identification and validation of four photodynamic therapy related genes inhibiting MAPK and inducing cell cycle alteration in squamous cell carcinoma
Source: Front Oncol. 2022 Aug 4;12:946493. doi: 10.3389/fonc.2022.946493 (PMC9386316; doi:10.3389/fonc.2022.946493)

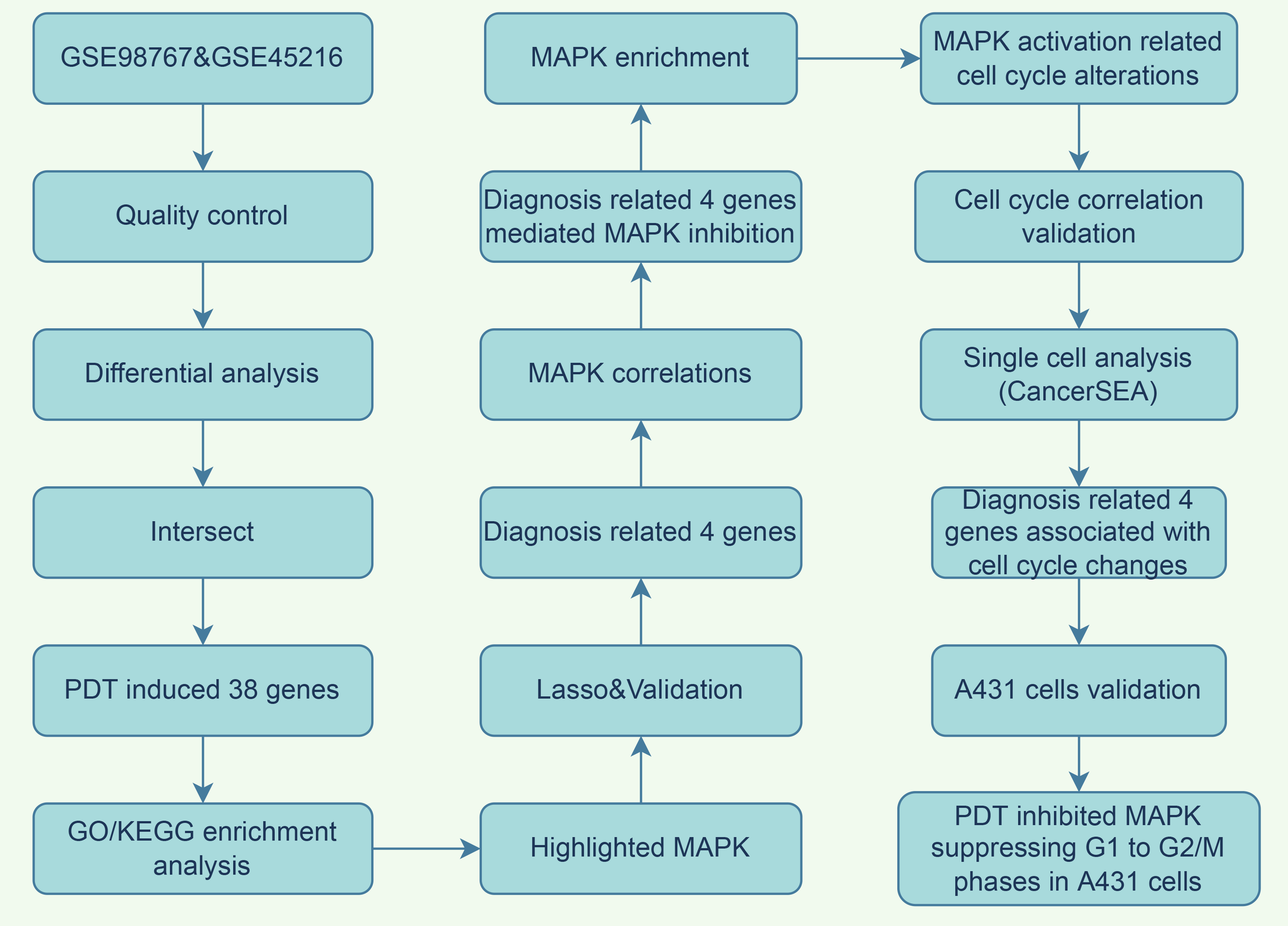

Supplement: Supplementary Figure 1 — The study design identified 4 potential targeted genes after PDT treatment regulating MAPK activation and cell cycle in cSCC. [file Image_1.tiff]

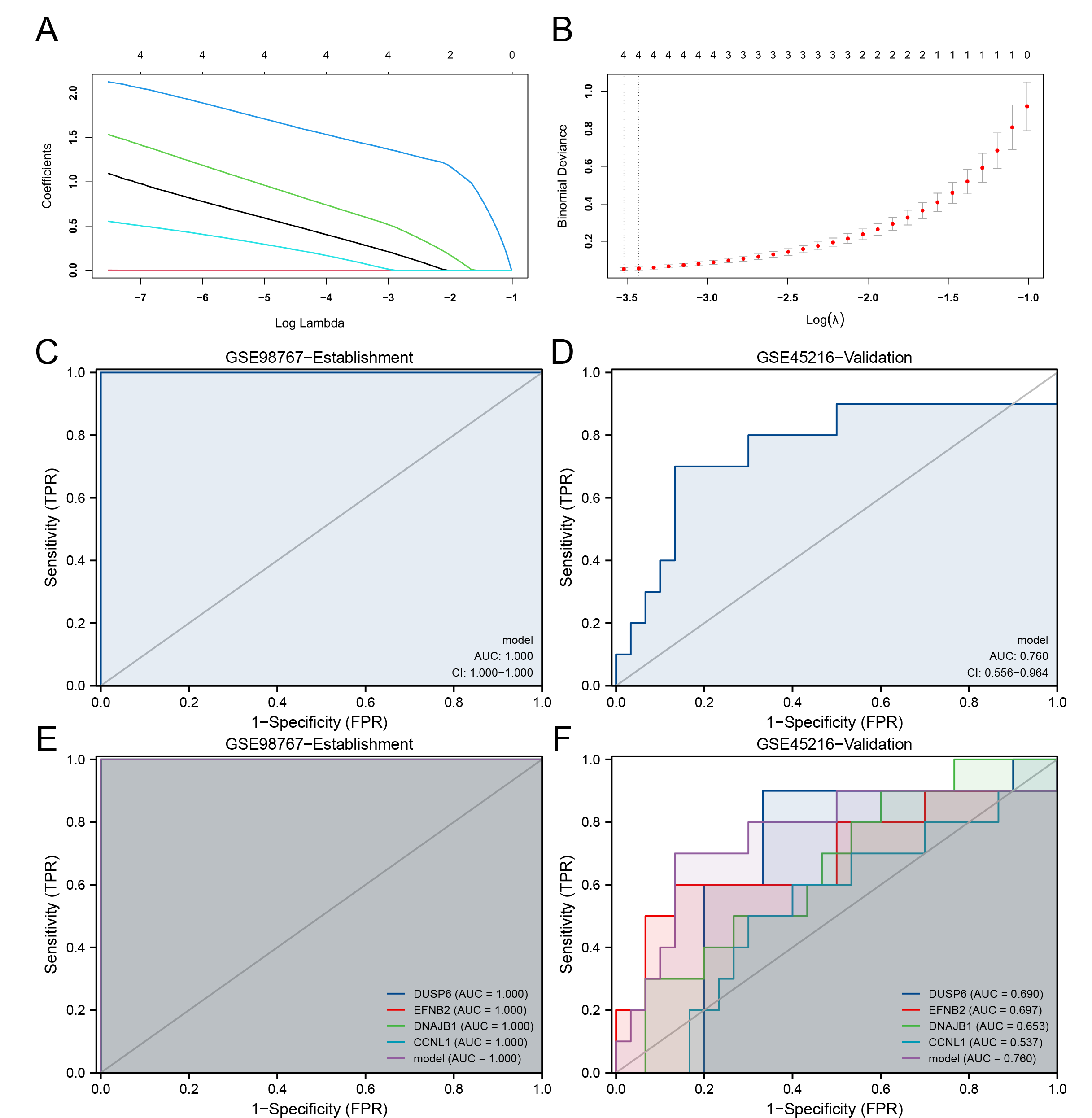

Supplement: Supplementary Figure 2 — Establishment and validation of diagnosis model by Lasso regression and ROC curve. (A) Coefficient profiles of 38 cSCC-PDT-related genes. (B) Selection of the optimal lambda in the Lasso model. (C–F) ROC curve of diagnosis model and expression level of above 4 genes. [file Image_2.tiff]

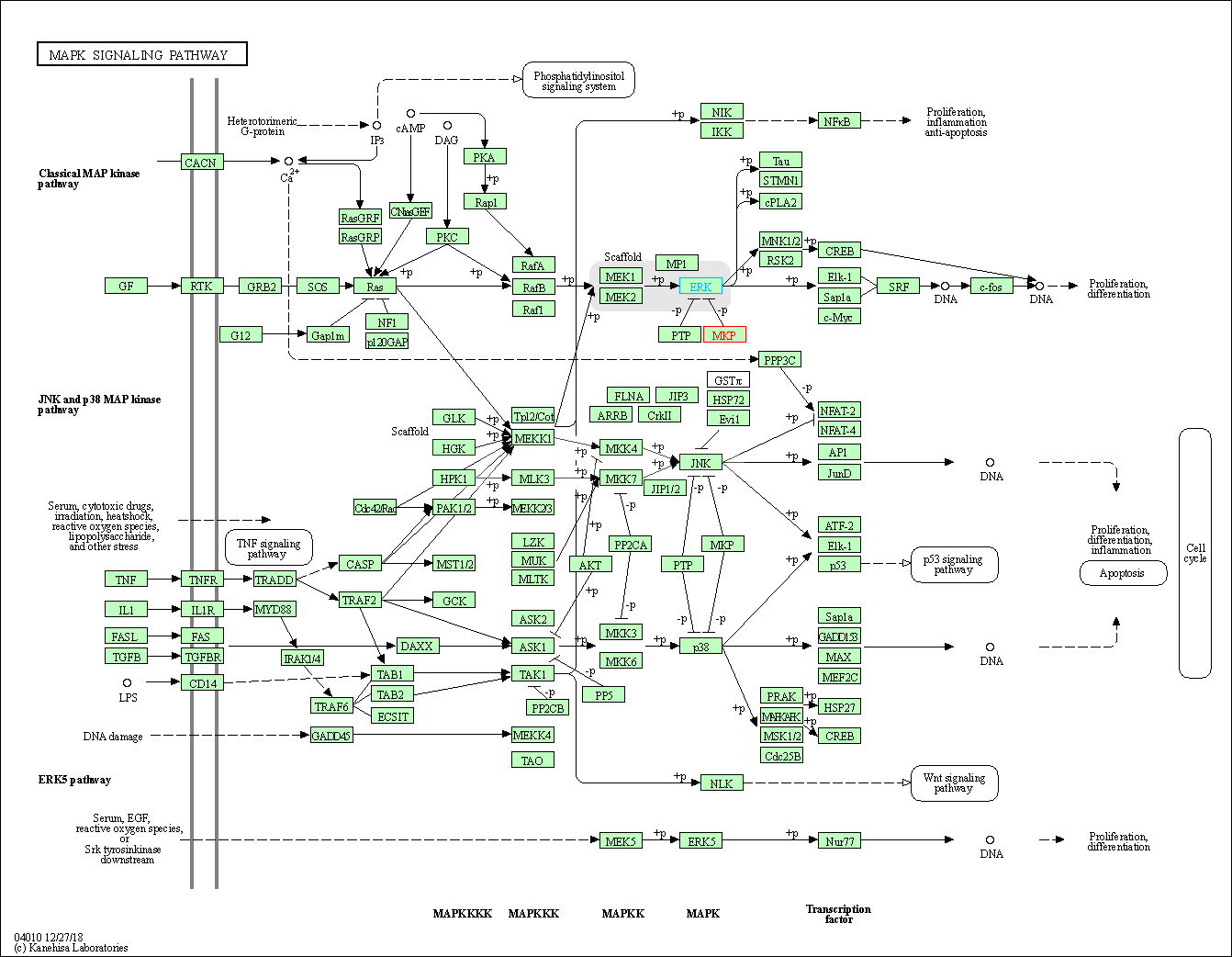

Supplement: Supplementary Figure 3 — KEGG pathway map of MAPK signaling pathway (hsa04010, https://www.genome.jp/pathway/hsa04010+1848) [file Image_3.tiff]

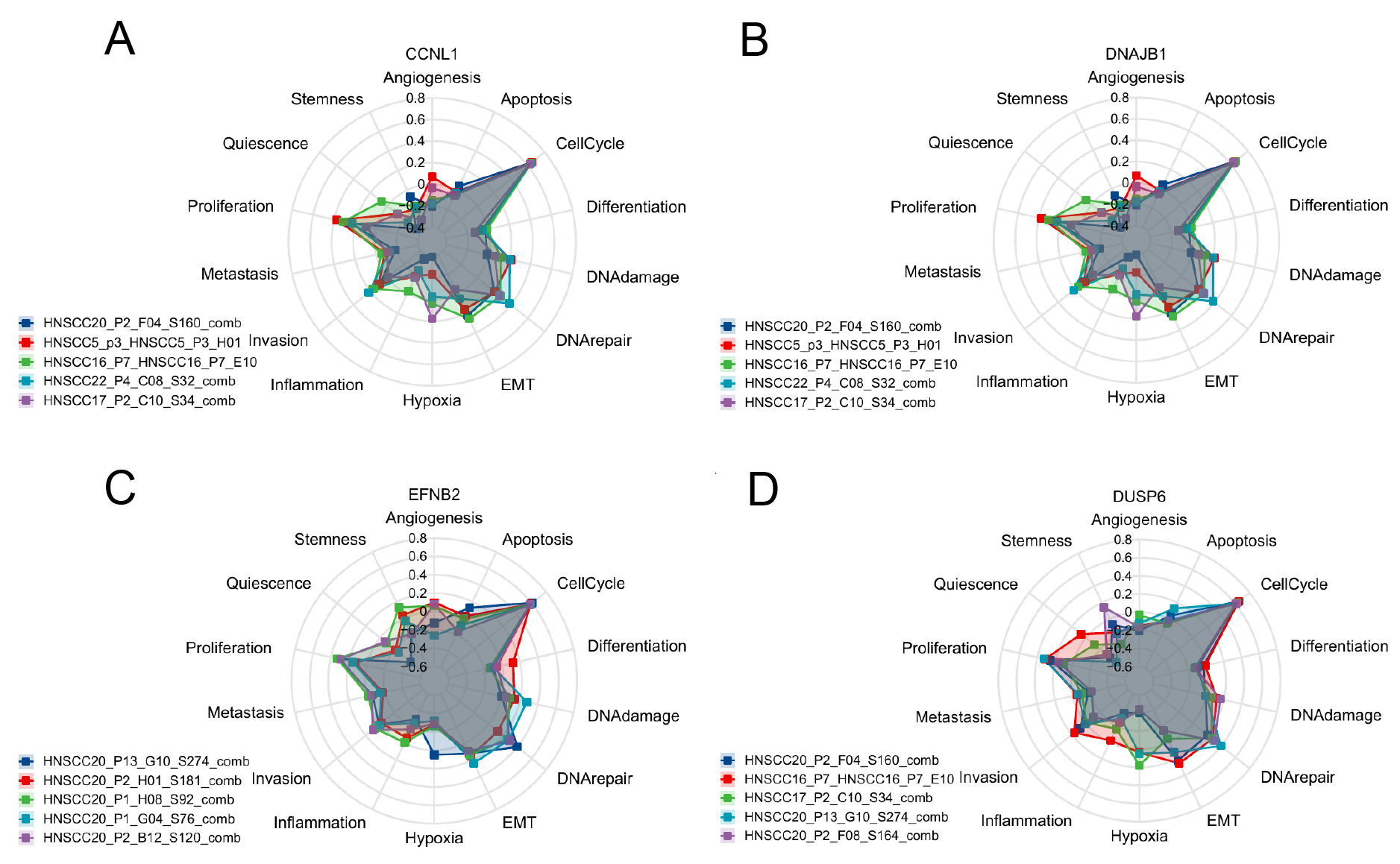

Supplement: Supplementary Figure 4 — Radar plot of top five single cells with high cell cycle function state after comprehensively ranked by gene expression level and cell cycle function state. (A–D) The single cell set was sequentially sorted by expression levels of CCNL1, DNAJB1, EFNB2 and DUSP6. [file Image_4.tiff]
